# The role of geometry in morphogenesis

**DOI:** 10.1242/dev.205774

**Published:** 2026-08-28

**Authors:** Ryan Harrison, Olivier Hamant, Timothy E. Saunders

**Affiliations:** ^1^ Warwick Medical School, University of Warwick, Coventry CV4 7AL, UK; ^2^ The Francis Crick Institute, 1 Midland Road, London NW1 1AT, UK; ^3^ Laboratoire de Reproduction et Développement des Plantes, ENS de Lyon, INRAE, CNRS, 46 Allée d'Italie, 69364 Lyon Cedex 07, France; ^4^ Centre for Mechanochemical Cell Biology, University of Warwick, Coventry CV4 7AL, UK

**Keywords:** Morphogenesis, Geometry, Boundaries, Mechanical feedback, Organogenesis

## Abstract

Across multicellular life, cells need to act in a coordinated manner to generate tissues with specific and reproducible forms necessary for their effective function. This process of morphogenesis is regulated by a myriad of signalling and mechanical inputs, which are often inter-related. Tissue morphogenesis typically occurs within constrained environments, such as the eggshell or buds for plants, that often have complex geometries. Yet, understanding how such boundaries impact the formation of tissues and organs has remained challenging. Recent advances in imaging, *in vitro* methods and genetic manipulation are leading to new understanding of how geometry interplays with signalling and mechanics to ensure tissues form robustly. Here, we focus on the role of geometric constraints in guiding morphogenesis, utilising examples from across the animal and plant kingdoms to highlight key principles. This demonstrates that geometry can be instructive, rather than simply a passive constraint, during morphogenesis.

## Teaser

**Summary:** This Review looks at the role of boundaries in morphogenesis, with a focus on how such boundaries can be instructive in morphogenesis and not just a passive constraint.

## INTRODUCTION

In a given multicellular organism, organs have reproducible shapes and sizes necessary for their function (Hamant and Saunders, 2020). Organs are comprised of multiple cell types that must function together precisely and robustly (Lecuit and Le Goff, 2007). The generation of shape – morphogenesis – has been an ongoing subject of study since antiquity, when Aristotle identified conserved structures across species and suggested they may serve similar functions (Blits, 1999). The beginning of the 20th Century saw attempts to mathematically describe how biological form emerges (Haldane, 1926; Saunders and Ingham, 2017) and how animals reach the ‘right size’ for effective function (Haldane, 1926). Yet, our understanding of how complex tissue and organ structures emerge in a robust way is still incomplete today (Ingber, 1993). This is, in part, because of several interesting problems and trade-offs. First, increased geometric precision through increased developmental control may hinder robustness (and vice versa). For example, precision can be achieved by a high degree of top-down control, but that strategy is inherently fragile as it depends on control efficiency. Conversely, shape reproducibility frequently emerges from variable cell behaviour because bottom-up emergence is typically more robust as it accommodates, and even builds on, inherent variability (or ‘noise’) present in biological systems. Last, while we as experimentalists typically observe mean properties (e.g. lung volume), cells are more perceptive to the local standard deviation (e.g. fluctuating signals).

Recent technological advances have taken morphogenesis to new spatio-temporal resolutions. Single-cell sequencing enables us to probe individual cell states and differentiate cell populations within developing systems (Baysoy et al., 2023). Spatial-omics approaches even enable information about the cell proteomic, transcriptomic and chromatin states to be accessed and directly related to the physiological position (Bodenmiller, 2024; Neumann et al., 2022; Wan et al., 2026). However, chemistry does not explain geometry (Box 1); understanding cell identity at a single cell scale alone is insufficient to explain how complex form emerges. We emphasise that even if we knew the complete molecular state of a group of cells, we would not be able to predict the shape of the resultant tissue without knowledge of the acting forces, boundary effects and external stressors (Box 1).

**Fig. 1.**
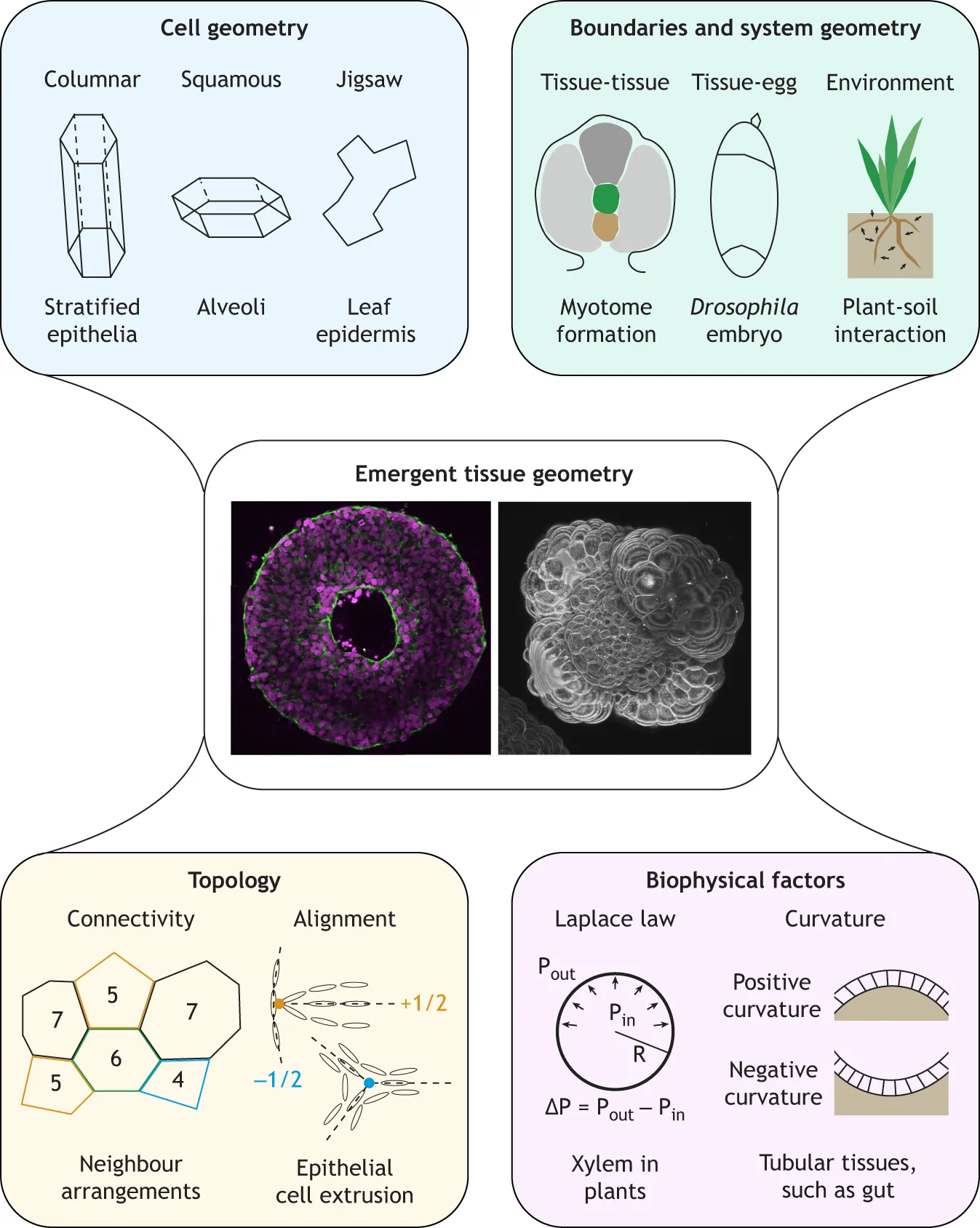
**Role of geometry in shaping tissues.** Multiple factors, including cell shape (top left), boundary constraints (top right), topological inputs (bottom left) including from different cell alignment defects (±1/2) and biophysical factors (bottom right) impact how the final tissue form emerges (centre). Centre shows images of a 48 h neuruloid (left; magenta, DAPI; green, actin) and a young *Arabidopsis* flower (right) treated with oryzalin (depolymerised microtubules) thus showing isotropic growth, but no defect in patterning (organs are still initiated in the right position).

Box 1. Defining shape The concept of shape is important across disciplines, from the structure of atoms through to the form of particular organs. The language to describe shape varies across fields and, as such, can lead to confusion. Here, we define the key conceptual terms around shape with regards to how we use them in this review (Fig. 1).

**Geometry.** What shape does an organ/tissue have? Here, we consider geometry as defining the size (e.g. volume), shape (e.g. skew, twist, etc.), curvature and spatial arrangement of cells and tissues in defining embryonic structures. Cell/tissue geometry is impacted by mechanical forces, boundary constraints, signalling inputs, packing constraints and growth. For developmental systems, we often have to consider geometries at different scales. The specific cell geometry is defined by the local forces, both intrinsic to the cell and from interactions with neighbours. At larger scales, the developing tissue may be subject to long-ranged, supracellular forces that depend on the shape of the organ.

**Boundaries.** Where do cellular/tissues domains begin and end? Here, we refer to boundaries as the physical interfaces separating distinct tissue domains. They can be defined by differences in cell identity, mechanical stiffness, adhesion molecules, extracellular matrix composition or signalling activity. In both plants and animals, boundaries restrict or guide cell movement, maintain compartment identity and act as organising centres for signals. Crucially, during many developmental processes these boundaries are dynamic, and changes in boundaries can play an important role in tissue shaping. In other cases, boundaries can be defined by rigid structures, such as egg-shells, for example, in *Drosophila* embryogenesis. Here, we refer to such larger scale constraints that encompass both a tissue and its surrounding environment as ‘system geometry’.

**Topology.** How connected are different cells/tissues? Whereas geometry describes the local form of the cells/tissues, topology represents the global organisation and interaction network of cells, which persists even if local geometry changes. Recent work has revealed how cell interconnectivity – and associated defects – can impact cell behaviour in tissues (Petridou et al., 2021; Saw et al., 2017). Topology is not our primary focus here, but it is important to recognise that topology and geometry are distinct (Gibson and Gibson, 2009).

**Anisotropy.** This describes the structural property of non-uniformity in different directions, as opposed to isotropy. Growth is anisotropic if a dominant direction of growth can be identified.

**Laplace–Young law.** For a pressurised system, tension and curvature are related by Laplace–Young law: Δ*P=2γ/R*, where Δ*P* represents the pressure difference across the surface, γ is the surface tension and *R* the system radius.

**Curvature.** This defines the geometry of a sheet (e.g. a cell layer). Its value at a specific point in the sheet is proportional to the inverse radius of the fitting sphere at that point; i.e. a flat sheet has zero curvature as this corresponds to an infinitely large sphere. Curvature has two principal components, and these can be positive (e.g. a sphere) or negative (e.g. at a saddle) (Fig. 1). Gaussian curvature is defined by the product of these two principal curvatures. Cells can sense local curvature, and we discuss examples of how this can guide tissue development in the main text.

To understand how multicellular architectures are built, we also require knowledge of mechanical factors behind shape emergence (Fig. 1), such as oriented forces that change cell shapes and generate long-ranged structures (Green, 2022; Hamant and Saunders, 2020; Heisenberg and Bellaïche, 2013; Lecuit and Le Goff, 2007). These forces act across spatial scales, from subcellular processes through to whole tissue remodelling (Anlas and Nelson, 2018). For example, actomyosin contractility can drive oriented cell rearrangements that lead to tissue elongation in *Drosophila* (Collinet et al., 2015), and co-alignment of cortical microtubules directs growth anisotropy (Box 1) through oriented cellulose deposition in plant cells (Paredez et al., 2006). Crucially, mechanics and cell genetic state are coupled (Bailles et al., 2022; Collinet and Lecuit, 2021); mechanical processes are not only downstream effects of specific gene regulation, but can themselves impact gene regulation (He et al., 2026; Hervé and Miroshnikova, 2024; Leckband, 2023; Vignes et al., 2022). In the developing chick embryo, feedback between mechanical forces and tissue patterning ensure robust self-organisation of the epiblast (Caldarelli et al., 2024). Similarly, in *Arabidopsis*, mechanical conflicts emerging from differential growth in sepals can explain how floral buds remain locked before anthesis (Trinh et al., 2024). However, manipulation of *Drosophila* egg size has revealed the limitations of patterning and mechanical processes in generating precise form (Horne-Badovinac et al., 2012; Huang et al., 2020); knowledge of the system geometry (the geometry of both a specific tissue and its surrounding environment; Box 1) is also necessary.

Physical boundaries (Box 1) have long been appreciated as important in tissue morphogenesis, especially related to biochemical signalling, e.g. from morphogens (Espina et al., 2023; Kicheva and Briscoe, 2023). Morphogens are often secreted at the interfaces between neighbouring tissues, providing spatial information to developing systems, including in regulating scaling (Amourda and Saunders, 2017). Boundaries also provide geometric constraints to developing tissues, such as in animal eggshells, and can act as instructive geometric cues (Stoddard et al., 2017). This is self-explanatory in plants: the boundary between a leaf and a stem always hosts a bud, and thus acts as a provider of hormones such as auxin, a key driver of plant architecture (Wang et al., 2018). Cross-species comparisons have shown how changes in boundary constraints can be compensated for by, for example, altering system dynamics (Gregor et al., 2008; Kicheva et al., 2014). Such compensation is prevalent in plants (Horiguchi and Tsukaya, 2011); however, it remains challenging to perturb geometry and boundaries, especially in vertebrate systems, thus limiting our understanding of their role in directing morphogenesis. The local environment around cells and tissues can create physical interfaces, such as long-ranged actomyosin cables (Balaghi et al., 2023), composition and stiffness of surrounding extracellular matrix (ECM) (Díaz-de-la-Loza and Stramer, 2024), pressure from neighbouring tissues (Espina et al., 2023; Tlili et al., 2019) and restriction by extra-embryonic tissues (Panfilio and Chuva de Sousa Lopes, 2022) or other protective layers (Couturier et al., 2009). Further, system geometry often changes across time, such as during vertebrate body axis extension (Benazeraf and Pourquié, 2013; Stooke-Vaughan et al., 2025) or lateral root growth (Vilches-Barro and Maizel, 2015). Yet, despite substantial work, the role of geometry remains a poorly characterised component of morphogenesis.

In this Review, we highlight how system geometry is actively involved in guiding morphogenesis. We begin by focusing on the relationship between cells, tissues and their local environments. We then survey systems undergoing significant growth during organ formation and how boundary feedback is important in guiding morphogenesis. We also discuss how the system geometry impacts cell migration during organ formation, e.g. in the heart. We then turn our attention to new *in vitro* models of organogenesis, where geometry can be more precisely controlled, before finally reviewing theoretical approaches for understanding how feedback between boundary conditions and developing tissues ensures robust morphogenesis. Overall, we present a multi-scale, multi-kingdom perspective on how geometry impacts development and highlight exciting new methods that we believe will reveal new insights into morphogenesis.

## Tissue interactions with the local environment

In this first section, we discuss how specific cell and tissue shapes emerge during development (Fig. 1). To this end, it is important to highlight how cells sense and respond to geometric constraints, both locally and from the tissue scale environment. Here, we focus on the ECM and adhesion as important factors coupling these different components. We consider adhesion in a broader sense, including both cell-ECM and cell-cell (e.g. Cadherin-mediated) adhesion, and detail here the interactions between cells and geometry instead of specific molecular mechanisms.

### The role of ECM in shaping tissues

Animal cells interact with their neighbourhood through the local ECM, which can be deposited by the specific tissue, its neighbouring tissues or even cells migrating through the local domain (Brown, 2011). Interactions between cells and the ECM can be finely tuned through adhesion molecules, which change the physical nature of the tissue (Díaz-de-la-Loza and Stramer, 2024; Elosegui-Artola, 2021). Such interactions typically occur within defined boundaries, which can have geometries ranging from flat, to curved or buckled. This, in turn, can impact morphogenesis; we discuss three examples from *Drosophila* below that highlight different aspects of this behaviour. Of course, the interplay between ECM, geometry and tissue shape has been explored in many systems (Keller, 2012), such as zebrafish eye formation (Soans et al., 2022) and mesendoderm morphogenesis during *Xenopus* gastrulation (Davidson et al., 2002). Our chosen examples highlight how curved environments in particular can impact cell and tissue shape.

**Fig. 2.**
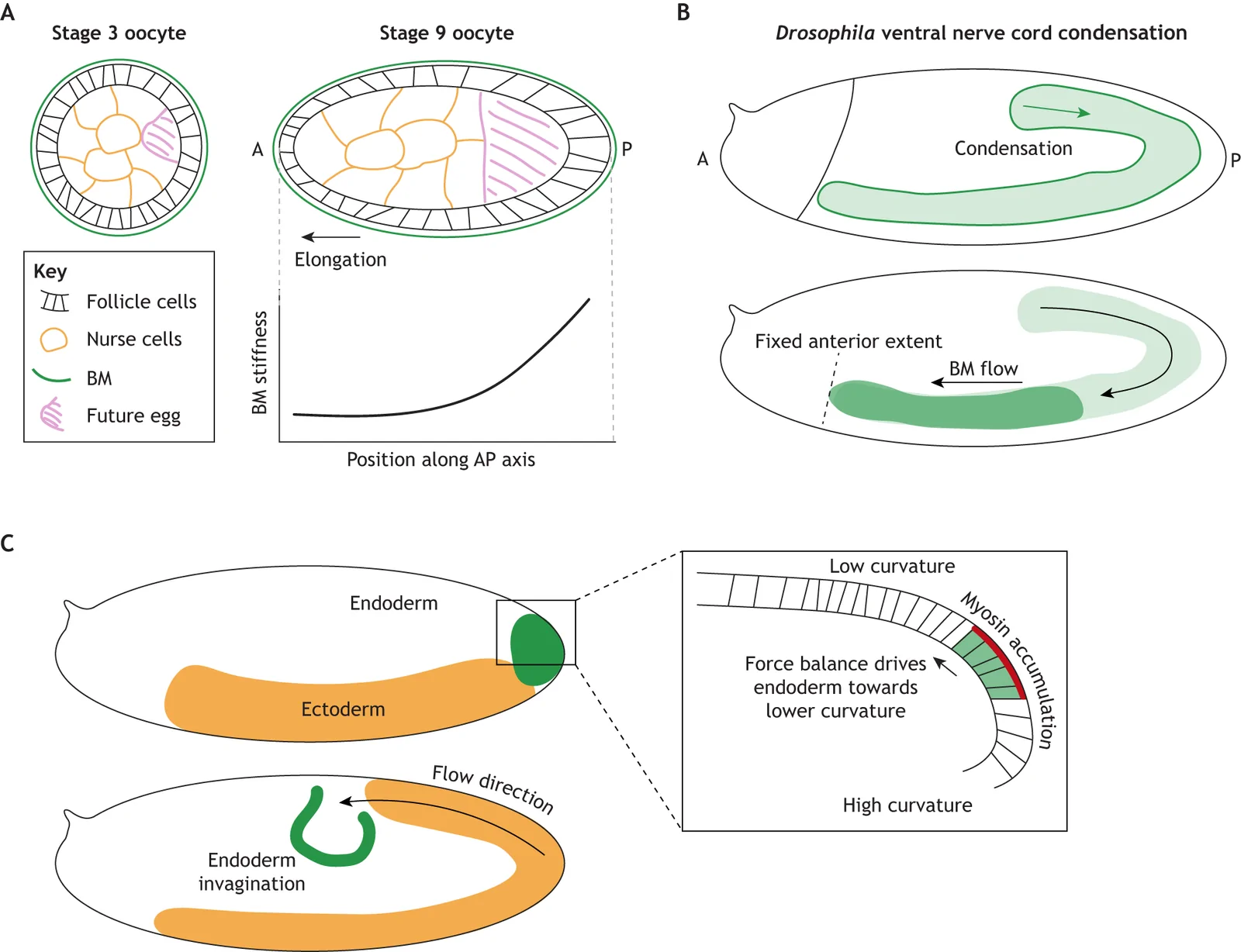
**Coupling between tissues and surrounding boundaries directs morphogenesis.** (A) *Drosophila* follicle elongation. At early stages (stage 3), the oocyte is spherical and the future egg has a spherical geometry. At later oocyte development (stage 9), a gradient of basement membrane (BM) stiffness drives elongation of the oocyte to generate an ellipsoidal geometry along the anterior-posterior (AP) axis. (B) *Drosophila* ventral nerve cord condensation. Top: a fully elongated nerve cord at around stage 11 of embryo development. Bottom: the reduced size (dark green) of the nerve cord by stage 16 (near to hatching). The nerve cord condenses from the posterior, with little change in the anterior extent. The distribution of BM changes during morphogenesis. (C) Movement of the endoderm in the early *Drosophila* embryo depends on the local embryo curvature through mechanical feedback with local myosin activity.

An ECM component that is crucial for the morphogenesis of multiple tissues is the basement membrane (BM) – a thin, planar-organised ECM that underlies the basal surface of epithelial tissues. The organisation of the surrounding ECM can guide morphogenesis. An example of this is *Drosophila* follicle formation (Fig. 2A), which involves reshaping of the tissue from a sphere into an elongated ellipsoid (Haigo and Bilder, 2011). This reshaping depends on finely tuning ECM stiffness and the orientation of its fibres, which can also vary across the tissue (Crest et al., 2017). Shaping of the follicle is driven by re-orientation of its anterior cells in response to cues from the underlying BM, which increases the local curvature (Chen et al., 2019). Mechanochemical feedback via Src tyrosine kinase – which is dependent on BM stiffness – enables robust reshaping of the follicle from round to an elongated structure. It is important to note that BM stiffness is not constant across the follicle (Fig. 2A). BM softening around cells that flatten out at the surface near the highly curved anterior and posterior poles alters the local curvature (Chlasta et al., 2017), which can potentially play a role in directing cell movements, a process known as ‘curvotaxis’ (Pieuchot et al., 2018; Vassaux et al., 2019). Follicle formation provides an example of how a developing tissue generates its own boundary constraints that then feedback into morphogenesis, further altering the system geometry.

In the *Drosophila* embryo, the ventral nerve cord (VNC) undergoes condensation, reducing in length by over 50% from its full extent over a period of 12 h during embryogenesis. The initial VNC is elongated and curves around the embryo posterior (Fig. 2B). However, by the end of condensation it forms a (largely) flat tissue along the dorsal axis of the embryo. This occurs within a highly constrained, curved environment along the embryo anterior-posterior axis. How does the tissue condense within a curved boundary? Two processes are essential in the tissue reshaping. First, Collagen IV increases in the surrounding ECM during condensation, which increases local tension and drives VNC condensation (Serna-Morales et al., 2023). Secondly, recent *in toto* imaging studies on the process of condensation has revealed that the neural and glial cells forming the VNC undergo significant shape changes that adapt as the tissue condenses (Karkali et al., 2022, 2023). Together, these processes ensure robust condensation of the tissue to its final shape along the dorsal region of the embryo, despite large-scale morphological changes (e.g. more than halving in length) within varying geometric constraints as the VNC condenses around the highly curved embryo posterior (Fig. 2B).

Finally, we consider how interactions between cells and ECMs form a curved tissue shape in a growing tissue. Eye formation has proven to be an excellent system to understand the interplay between geometry and ECM in sculpting a growing organ. In *Drosophila*, the spherical compound eye forms with precisely defined cell shapes that depend on the underlying ECM (Pichaud and Casares, 2022; Walther et al., 2024). This interaction is mediated by integrins, adhesion molecules that coordinate cell mechanics with the surrounding ECM. Through intricate cell-specific tuning of cell-ECM interactions, the curved insect compound eye can be built reliably (Lancaster et al., 2025; Pichaud and Casares, 2022). We discuss the feedback between adhesion and tissue geometry further below.

### Geometry, integrin-mediated cell adhesion and friction in animal morphogenesis

Morphogenesis typically involves large-scale cell movements. As cells move, they experience friction with the underlying substrate, mediated by the ECM and associated adhesion molecules (Smutny et al., 2017). Careful balancing of friction forces with intrinsically generated cell forces ensures precise positioning of cells and subsequent robust tissue morphogenesis. These processes often take place within geometrically confined environments, which provide spatial information to the forming tissues through mechanical constraints. For example, invagination and migration of the posterior endoderm in *Drosophila* requires movement around the highly curved posterior pole, regulated through integrin coupling between the tissue and the egg boundary (Bailles et al., 2019). Indeed, the curvature of the egg boundary is crucial in driving polarised tissue flow through coupling with myosin, which ensures robust tissue movement (Gehrels et al., 2023). Moreover, mechanical feedback is dependent on the curvature of the embryo eggshell (Box 1; Fig. 2C), which drives the posterior endoderm dorsally. Similar behaviour is seen during gastrulation in the short germ-band flour beetle *Tribolium*, whereby adhesion to the vitelline membrane boundary controls the positioning and timing of gastrulation (Münster et al., 2019). In both these examples, directional cues from the curved geometry of the bounding eggshell are necessary to ensure robust tissue morphogenesis.

### Adhesion and friction in plant morphogenesis

As adjacent plant cells share a stiff ECM, cell contiguity is maintained. This means that the growth of a given cell – i.e. the geometry it takes on – is constrained by adjacent cells (Bassel et al., 2014). As such, the role of friction is often not considered pertinent as plant cells are typically considered static relative to their neighbours. However, looking more deeply reveals that this assumption is not always correct.

Angiosperms, for example, host cells that migrate within a stiff environment; this necessitates an environment of reduced adhesion and friction. In particular, pollen tubes grow within the wall of papilla cells in the stigma to then reach the style and ovary, where two male gametes fertilise the egg (giving rise to the embryo) and the central cell (giving rise to the albumen). In the *Arabidopsis katanin* mutant – where cellulose deposition is affected due to microtubule misalignment, pollen tube expansion is more permissive, leading to coiling of the tube around the papilla cell, thus showing that adhesion and friction can determine pollen tube shape (Riglet et al., 2020). More generally, while adhesion has long been considered a passive consequence of cell wall formation in plants, functional analyses of mutants with adhesion defects suggest that adhesion is instead an active mechanism, with important consequences for growth and development, notably through tissue integrity (Asaoka et al., 2021; Atakhani et al., 2022). Here the interplay between adhesion and the highly curved tube is required to guide organ formation.

Several cell wall integrity pathways have also been identified in plant cells (Wolf, 2022). This further demonstrates that in plants, as in animals or fungi, the ECM can act as an instructive signal for development and, therefore, that cells monitor the dynamics of their ECM (e.g. responding to tension, desequestration of molecules, production of secondary byproducts of local disruptions, etc.). Interestingly, the outer wall of the epidermis of aerial plant organs, which is usually load-bearing and mechanically reinforced, can be compared to the lamina of animal epithelia. In both cases, the mechanical and geometric microenvironment can instruct cell behaviour; in particular, the mechanical reinforcement and enforced adhesion under tensile stress (Malivert et al., 2018).

In this section, we have focused on the cellular interactions with the ECM and the role of molecular sensors and effectors in guiding tissue morphogenesis within geometrically confined systems. Scaling up, we next move to how the geometry defined by boundaries between tissues impacts morphogenesis.

## The role of geometric constraints in growing tissues

Tissues grow and change shape during morphogenesis. Furthermore, surrounding tissues and environments also adapt, meaning that these boundaries are often dynamic. Recent advances in *in vivo* imaging and quantitative approaches are enabling the interplay between dynamic boundaries and morphogenesis to be dissected. In this section, we highlight vertebrate body axis elongation and skeletal muscle formation in animals, as well as lateral root growth in plants to exemplify the inter-relation of geometry and growth.

### Vertebrate axis elongation

Early in vertebrate development, the anterior-posterior body axis is specified by a highly conserved, well-defined molecular signalling network (Li and Elowitz, 2019; Stower and Srinivas, 2018). Elongation of the body axis for amniotes occurs through the continuous addition of neural and mesodermal tissue to the caudal end of the developing embryo from a progenitor pool in the primitive streak (Wymeersch et al., 2021). Neuromesodermal progenitors emerge from this region and give rise to paraxial mesoderm, which later segments into somites, as well as neural tissue that forms the posterior neural tube (Garriock et al., 2015).

Recent work in chick and zebrafish has revealed that material transitions in the involved tissues play a key role in axis elongation (Narayanan et al., 2021). Using chick presomitic mesoderm (PSM) explants, it has been shown that volumetric expansion of the trunk structure drives elongation, which results from graded cellular motility along the anteroposterior axis (Michaut et al., 2025). This expansion requires hyaluronic acid (Chugh et al., 2022), which alters viscous properties of the ECM. Even though cell positions are disordered, the resulting tissue-level structure is still structurally rigid (Mongera et al., 2018).

In mammals, it was recently revealed that surface tissue mechanics govern axis elongation. Work in an organoid model – later verified in the mouse embryo – showed that axis elongation is guided by the presence of a supracellular ‘actin cap’ structure (Trani-Bustos et al., 2025 preprint). This surface network constrains the expansive forces of cell divisions laterally and subsequently drives elongation in a posterior direction. The principal forces driving mammalian axis elongation are generated by cell proliferation instead of convergent-extension cell rearrangements, where a tissue narrows along one axis while extending in an another, which is more typical of other vertebrates. Therefore, we see that tuning system material properties (through a variety of mechanisms) within geometrically constrained environments can generate large-scale tissue structures from initially disordered conditions.

### Geometry guides skeletal muscle shaping

**Fig. 3.**
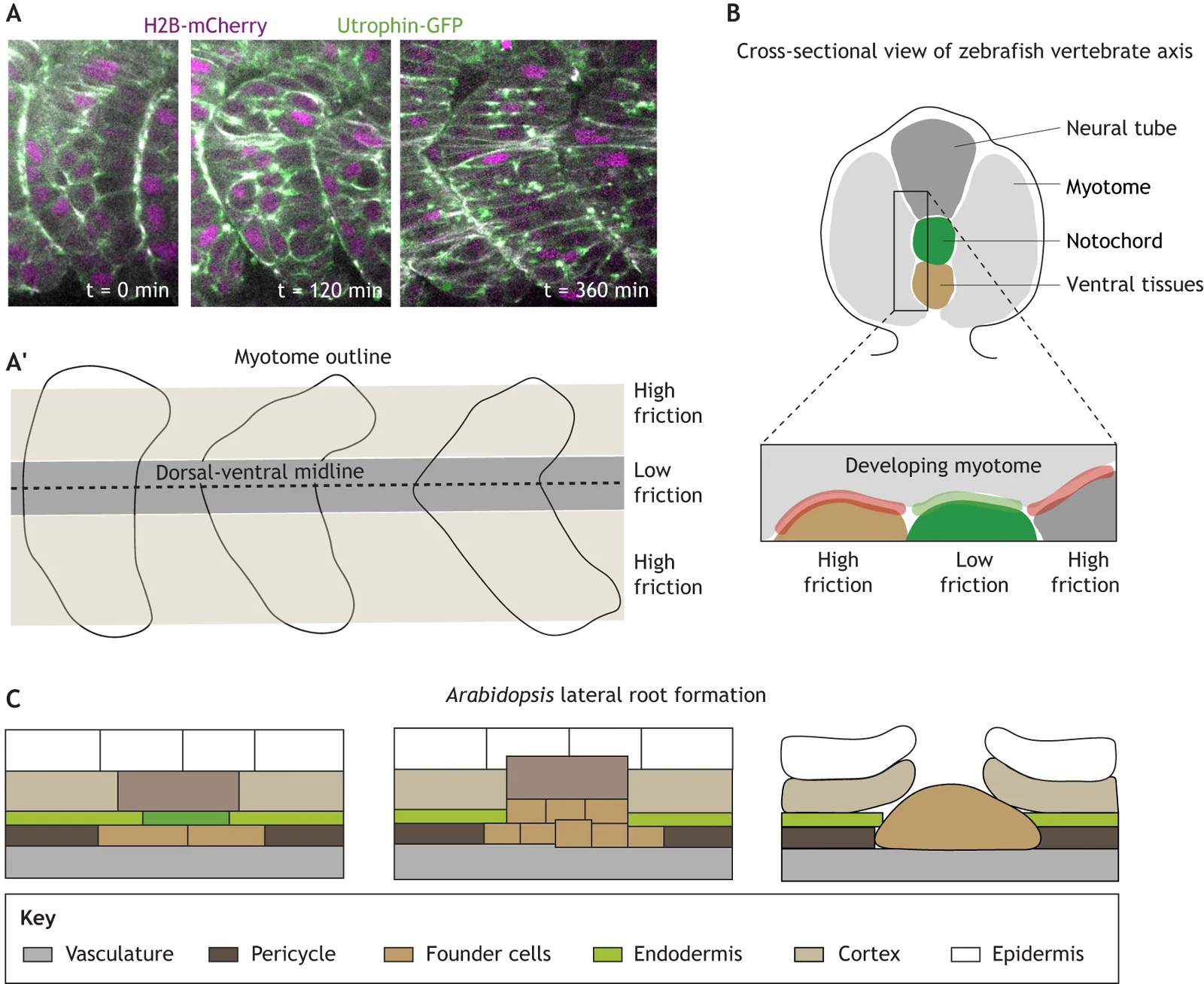
**Boundaries in growing tissues.** (A) Snapshots from a movie during formation of the zebrafish myotome. Time 0 corresponds to 24 h post fertilisation (hpf), somite 21. The actin rich domains defined by Utrophin-GFP denote the myotome boundaries. (A′) Outlines of future myotome segments shown in A, with the effective friction highlighted. (B) Cross-sectional representation of the developing myotome, highlighting neighbouring tissues. Interactions between the myotome and these neighbouring tissues generate differential friction that results in the distinctive chevron shape of the myotome. (C) Formation of lateral roots in *Arabidopsis*: high level of auxin in founder (LRP) cells (light brown) from the pericycle (dark brown) leads to their swelling and local thinning of the endodermis (green), local remodelling of the walls, crossing of the endodermis, cell separation of the epidermis (white) and cortex (beige/dark beige), and emergence of a lateral root.

In addition to establishing large scale order, environmental geometric constraints can guide the reshaping of tissues during development, as in zebrafish skeletal muscle formation. Here, future muscle segments (myotomes) emerge from somites and are reshaped into a distinctive chevron pattern (Fig. 3A) (Rost et al., 2014; Van Raamsdonk et al., 1979). This process is dependent on the interaction of the myotome with surrounding tissues (Tlili et al., 2019). Due to differences in ECM, the centre of the myotome experiences relatively less friction over the underlying notochord than with other tissues, such as the developing nervous system (Fig. 3B). This spatial gradient of frictions generates a shear force that is important for shaping the forming muscle into chevrons (Fig. 3A′) (Tlili et al., 2019), which is then ‘locked in’ by elongating muscle fibres (Mendieta-Serrano et al., 2022). Recent work has suggested that the chevron shape may already have been primed in the preceding presomitic mesoderm and induced by ordered cell arrangements in the dorsal and ventral domains of the tissue near boundaries with neighbouring tissues (Wang et al., 2026). Thus, geometric constraints from neighbouring tissues can guide complex tissue reshaping.

### Boundary constraints in lateral root growth

In plants, lateral root emergence is a coordinated mechanical and developmental process that allows the lateral root primordium (LRP) to cross the rigid endodermal barrier (Fig. 3C). The main constraint in this process is geometrical, as the endodermis is a stiff, continuous cylindrical layer surrounding the pericycle through which LRPs emerge. To go through the barrier created by the endodermis, the LRP generates an outward pushing force through turgor pressure, where high cytoplasmic volume causes the plasma membrane to push up against the cell wall. This growth produces directional mechanical pressure, but force alone is not sufficient to penetrate the endodermis; successful emergence also requires the endodermis to actively cooperate.

A key step is local remodelling of endodermal cell walls**.** Auxin produced by the LRP accumulates in the overlying endodermal cells, triggering the expression of wall-loosening enzymes such as expansins and pectin-modifying proteins (Lee and Kim, 2013; Lee et al., 2013; Lewis et al., 2013; Swarup et al., 2008). At the same time, endodermis integrity is transiently reduced, making the wall more flexible. The endodermal cells then partially separate, creating a zone of reduced mechanical resistance and changing the geometry of the cellular domain (Kumpf et al., 2013). While local cell death promotes LRP emergence (Escamez et al., 2020), such controlled softening allows the primordium to push through without rupturing the tissue; the tissues can adapt their geometry to the emerging LRP.

The endodermis also undergoes a temporary identity shift. Transcriptional regulators that normally maintain endodermal differentiation are locally suppressed. As a result, endodermal cells lose some of their barrier characteristics and adopt a more permissive state. The controlled reduction of endodermis volume serves as a cue for lateral root emergence (Vermeer et al., 2014), illustrating how geometry can also act as an instructive cue, in coordination with mechanics. The ‘bypass’ identity of the endodermis is reversible: once the LRP passes, endodermal features are restored. Hormonal crosstalk fine-tunes this process. Auxin is the primary biochemical signal coordinating LRP growth with endodermal yielding, while ethylene promotes cell separation and abscisic acid can strengthen the endodermis and inhibit emergence (Ding et al., 2015; Lewis et al., 2011).

Altogether, osmotic conditions, mechanical forces, wall modifications and identity changes create a controlled pathway through which the lateral root can make its way through a stiff tissue, while preserving overall root integrity (Fig. 3C).

## Geometry guides cell migration and tissue flows in animal organ morphogenesis

In contrast to most plant systems, where cells rarely engage in active movement, organ formation in animals is often characterised by significant cell migration and rearrangements. Such migration occurs within physical constraints that can be important for organogenesis. Alongside factors such as signalling gradients and physical properties of the substrate, the direction and speed of such migration can depend on the system geometry. Here, we provide an overview of how geometric constraints from boundaries impact cell migration. We use formation of the early heart as an example of how cell migration is impacted by the geometry of the surrounding environment, before considering how the system geometry impacts large-scale flows of cell collectives during tissue formation.

### The role of geometry in guiding cells during heart development

**Fig. 4.**
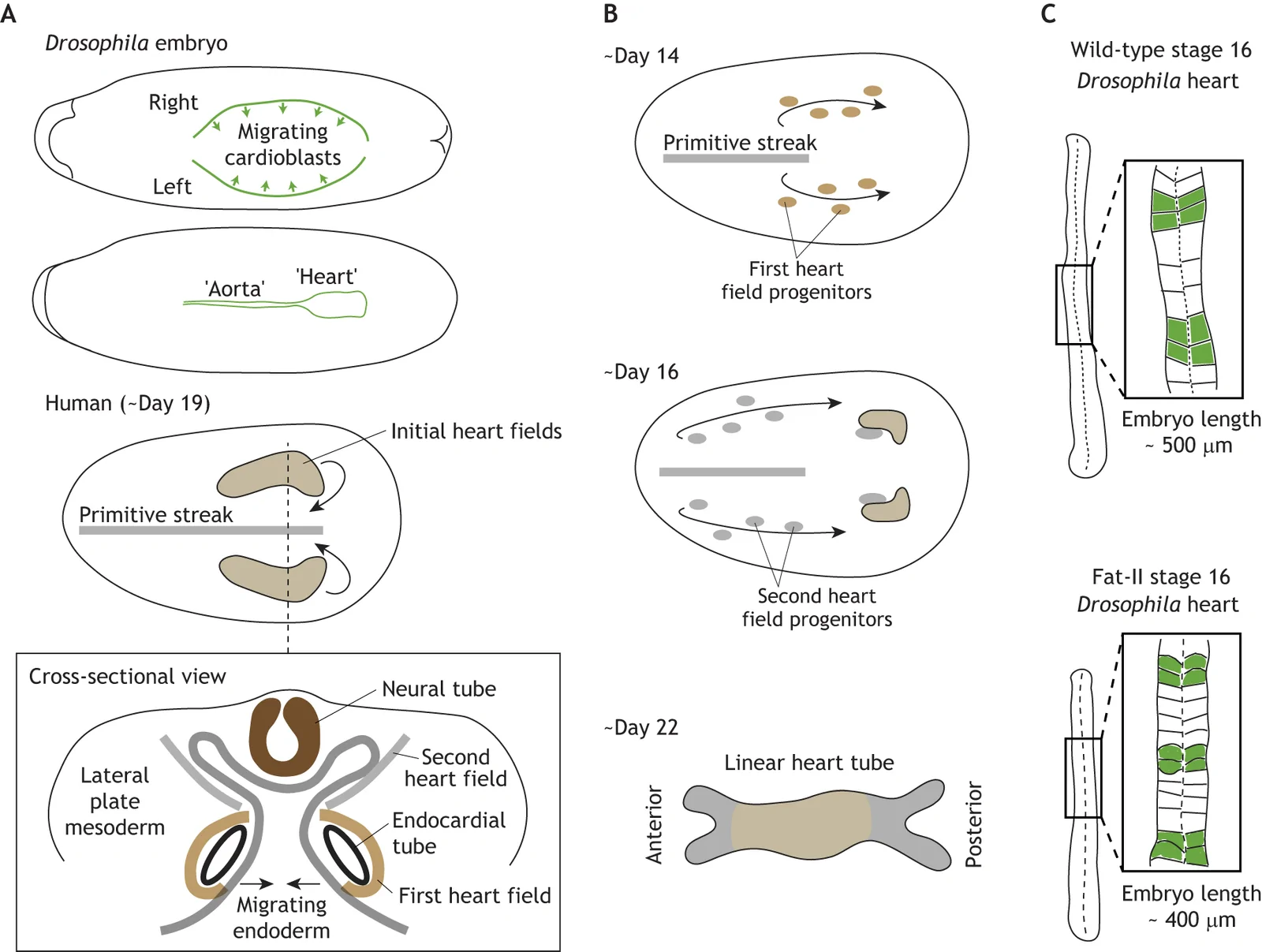
**Heart formation depends on boundary constraints and geometry.** (A) Formation of the *Drosophila* heart (top) and human heart (bottom) both require long-ranged migration with constrained environments. In *Drosophila*, the green areas denote the cardiac region from stage 14 (migration, top) and stage 16 (heart formation, bottom) are shown. Anterior to left. (B) The human heart forms from distinct waves of progenitor migration, that eventually lead to a linear heart tube at around day 22. (C) Altering the embryo size changes the length of the *Drosophila* heart by compacting cells along the anterior-posterior axis (anterior top), not by reducing cell number.

The heart is typically one of the first organs to form during animal development. The initial heart cells (cardioblasts) are specified by an array of different signalling inputs that interact through a highly conserved gene regulatory network (Olson, 2006). From flies to humans, early cardiogenesis involves the migration of two regions of mesoderm-derived cardioblasts towards each other to form a linear heart tube along the anterior-posterior axis of the embryo (Fig. 4A), confined by neighbouring tissues (including extra-embryonic tissues, such as amnioserosa in *Drosophila*) (Abu-Issa and Kirby, 2007; Jackson et al., 2017). This is an inherently mechanical process where differential adhesion (Santiago-Martínez et al., 2008; Zhang et al., 2018), actin localisation and contractility (Balaghi et al., 2023; Vogler et al., 2014; Zhang et al., 2018, 2020), ECM–cell interplay (Lockhart et al., 2011; Vanderploeg and Jacobs, 2015) and neighbouring cells (Zhang and Saunders, 2021) all impact early cardiogenesis (Buijtendijk et al., 2020). Failure in early heart formation can lead to a plethora of cardiovascular diseases (Bruneau, 2020).

Formation of the first cardiac structures occurs within the highly constrained embryo environment (Fig. 4A,B), for example by neighbouring mesoderm and endoderm tissues (Dominguez et al., 2023; McDole et al., 2018). These tissues are highly curved, providing a varied geometry at different locations for heart cells. How these cells reliably migrate within such an environment remains an open question. Interestingly, in size-reduced *Drosophila* embryos, the heart was able to scale with embryo size (Tiwari et al., 2021), suggesting that the forming heart cells can adapt to changing embryo geometry. This was achieved by the heart cells reducing their extent along the anterior-posterior axis, and not by altering cell number (Fig. 4C). A simple model of cell-cell interactions that favoured cell alignment as cardioblasts formed the initial heart tube was able to explain how cells are arranged under different initial geometric configurations and boundary constraints (Tlili et al., 2025). Recent work in zebrafish suggests that mechanical feedback can regulate heart morphogenesis to ensure that the heart reaches its final size (Andrews et al., 2025), but how the geometry of the neighbouring boundaries impact this process remains unknown.

### Role of geometry in guiding tissue flows

Cells within a tissue can migrate as a collective, generating effective flows through the developing organism. Such tissue flows are constrained by the geometry of the local environment. The movement of cells within such flows can be distinguished from single cell migration (as is the case for heart migration in mammals).

An example of tissue flow being impacted by geometry is the development of the *Drosophila* hindgut primordium (Keenan et al., 2022). Here, the structure begins as a ring of cells around the embryo posterior, which is reconfigured into a triangular ‘keyhole’ shape as the tissue migrates. By modelling the hindgut primordium as an inextensible elastic ring and applying geometric constraints from the ellipsoidal-like embryo, along with a stress associated with germband extension, such a keyhole architecture emerges without additional mechanical inputs; i.e. the boundary geometry alone is sufficient to instruct the shape of the tissue (Alber et al., 2025).

Germband elongation in the *Drosophila* embryo has long been a canonical system for understanding how mechanical forces guide morphogenesis, as the tissue doubles in length along the anterior-posterior axis while narrowing in the dorsal-ventral axis (Lecuit et al., 2011). Inducing local barriers by laser ablation alters tissue flow and subsequent elongation (Rauzi et al., 2015). Myosin-driven contractility initiates oriented cell rearrangements, which lead to germband elongation (Collinet et al., 2015). Recent work has proposed that geometric constraints from the curved embryo – which contains different levels of curvature in the dorsal (more curved) and ventral (less curved) domains – instructs myosin recruitment at different locations (Lefebvre et al., 2023). A simple mechanical feedback model based on this assumption was able to explain myosin distribution in a range of mutant phenotypes, including reduced-sized embryos. Such polarised mechanical forces direct embryo-scale tissue flows that play a crucial role in gastrulation.

Advances in quantitative approaches enable us to investigate how boundaries and geometry independently shape morphogenesis by directing tissue flows and cell migration across diverse systems (Krzic et al., 2012). Such data are revealing that geometry plays an essential role in morphogenesis by guiding tissue flows and cell migration. The system geometry can provide information to direct tissue shape along specific routes of morphogenesis and ensure symmetry breaking events occur robustly. Of course, there remain many open questions regarding how geometry influences cell and tissue dynamics, such as how oriented forces are initiated and maintained and how heterogeneity in cell mechanical and genetic state (Pillai et al., 2026) are compensated for by geometric inputs. Other model systems, such as migration in the neural crest (Barriga et al., 2018; Franze, 2013) and retina (Soans et al., 2022), along with *in vitro* models, provide exciting opportunities to explore these questions further.

## Tissue shaping within restricted environments

Most organs grow during development. Yet, the surrounding boundaries – whether these are environment (e.g. soil or outer shell) or neighbouring tissues – restrict such growth and such boundaries often impose complex geometric constraints (e.g. high curvature) on the growing tissue. Here, we consider a range of examples of how the environment impacts tissue geometry from both plant and animal systems.

### Plant response to external stress

Restricted environments play a crucial role in many aspects of plant development. For example, plant growth declines during drought partly because drying soil becomes mechanically stiffer, increasing soil compaction stress on roots. As water content drops, pore spaces shrink and soil mechanical impedance rises, restricting root elongation and reducing access to water and nutrients (Zhu et al., 2024). Compacted, drier soils also alter gas diffusion, causing ethylene buildup around root tips, which triggers growth arrest and further suppresses root system expansion (Zhang et al., 2026).

The aerial parts of plants are also under physical constraints. In particular, gravity, wind and water coherence determine the maximal height of trees (Slik et al., 2024). Beyond height, such constraint can also explain more complex geometric features. In particular, trees are self-similar structures: long and thick branches are near the ground, while short and thin branches are near the extremities. Wind and competition for light have been shown to play major roles in sculpting the architecture of tree branches, more so than hydraulics (Eloy et al., 2017).

Lastly, the emergence of lobed leaves in trees from temperate climates is a result of their development in a geometrically confined environment: the leaf bud. As the emerging leaves fill the available space inside the bud, they must fold and stop growing as they reach their geometric boundaries. When the bud opens, the leaf literally unfolds and reveals its lobed pattern (Couturier et al., 2011).

In this section, we have focused on how plant growth is controlled by external stress imposed by the environment and the impact this has on geometry. Of course, there are similar situations in animal systems, such as looping of the developing vertebrate gut (Savin et al., 2011). Next, we focus on recent advances in *in vitro* systems for both plants and animals, which provide methods to manipulate boundaries and more explicitly explore the role of geometry in morphogenesis.

### *In vitro* plant models for exploring the role of geometry in cell behaviour

**Fig. 5.**
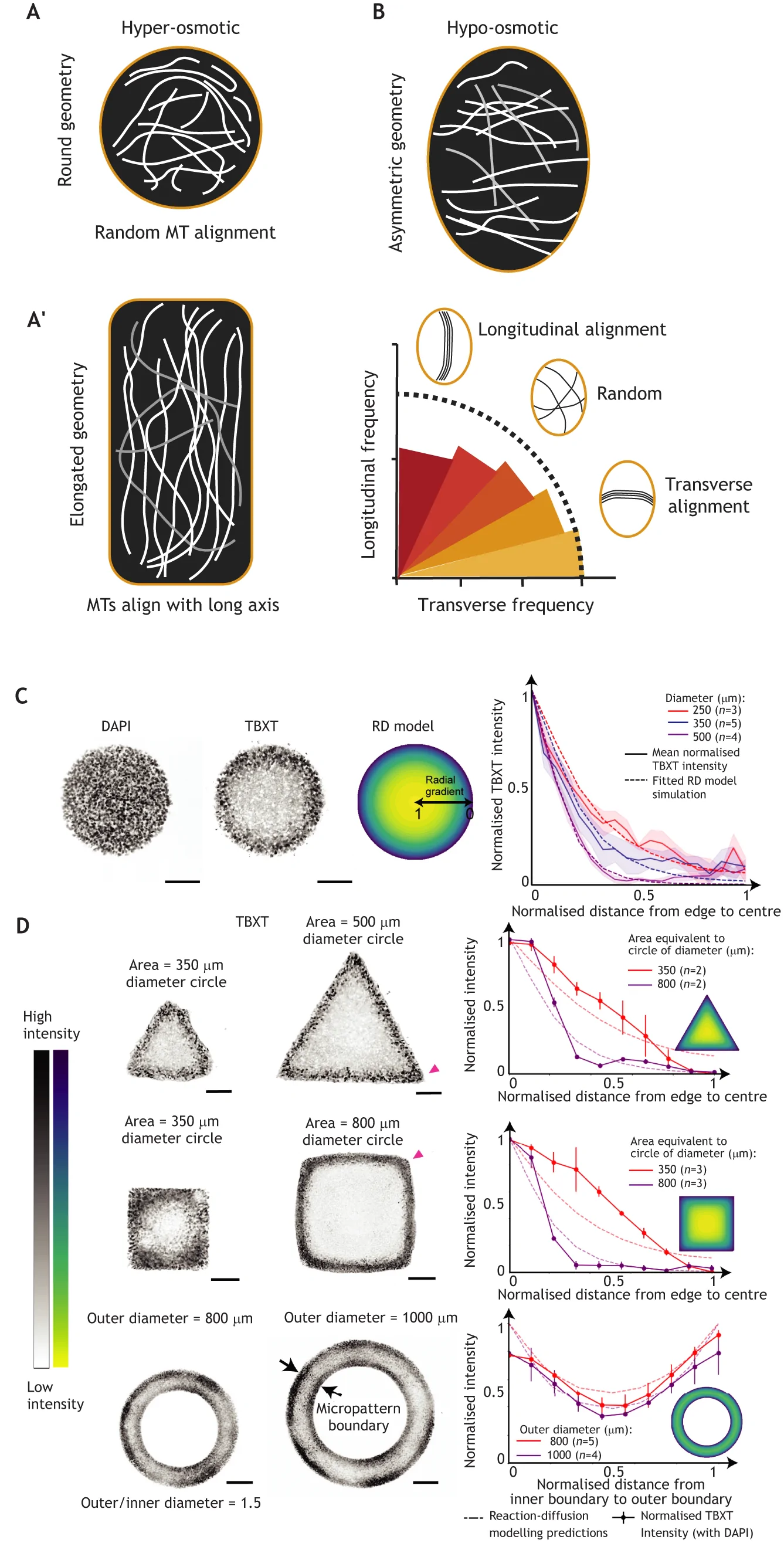
***In vitro* systems for exploring the role of geometry in morphogenesis.** (A,A′) Confined protoplasts in a hyper-osmotic environment display elongated polarised microtubule (MT) alignment (shown in white) with the long axis. (B) Confined protoplasts in a hypo-osmotic environment display a transverse bias in their microtubule orientation. Lower illustration highlights difference in alignment frequency between transverse and longitudinal directions. (C,D) TBXT patterning in neuruloids of varying geometry. (C) Intensities of DAPI and TBXT in circular neuruloids. A reaction-diffusion model is fitted to the experimental TBXT expression profile. (D) Different neuruloid micropatterns with fitted reaction-diffusion model. Importantly, the reaction-diffusion model can accurately predict the expression profiles in different boundaries simply by adjusting the geometric constraints. Panels A and B are adapted from Colin et al. (2020) and Durand-Smet et al. (2020) and panels C and D are adapted from Loo et al. (2026).

To monitor the impact of geometry on development, one can design moulds with specific shapes to test the impact on morphogenesis. A classic, and even commercial, example, is the square-shaped watermelon where the young fruit develops inside a rigid, square mould. This approach can also be applied in the lab, in more controlled conditions and at higher spatiotemporal resolution. For example, by confining protoplasts in microwells with defined geometries, one can turn spherical protoplasts (deformable, wall-less plant cells) into ellipsoidal protoplasts (Fig. 5A). One of the consequences of such geometric change is that the pattern of tension at the plasma membrane switches from isotropic to anisotropic, with maximal tension along the transverse axis, following the Laplace-Young law (Box 1). This property has been used to show that cortical microtubules orient with maximal tension direction, even in the absence of a cell wall, through geometry-derived mechanical patterns (Fig. 5B) (Colin et al., 2020). Interestingly, when placed in hyperosmotic conditions, tension decreases and cortical microtubules orient along the long axis of the protoplast. In that case, the high persistence length of microtubules instructs them to align along the flattest direction of the cortex*,* i.e. the long axis (Colin et al., 2020; Durand-Smet et al., 2020). Therefore, we see that the feedback between geometry and the cytoskeleton is complex and dynamic, and depends on, for example, local pressure (which also changes as the system geometry is altered).

Similar behaviour has been observed at tissue scales. For example, the compression of *Arabidopsis* hypocotyl (the stem immediately above the root of the embryonic plant) can trigger extra cambial cell files, which encircle the stem, in the region under compression, with division planes aligning with the direction of maximal tension (i.e. parallel to the compression force) (Höfler et al., 2024). Such approaches have been used on dedifferentiated plant tissues (Sugimoto et al., 2010; Varapparambath et al., 2022): compression also induces division plane alignment along maximal tension (Lintilhac and Vesecky, 1984).

Without external deformation, tissue shape can also act as an instructive cue, with important developmental implications. For instance, cylindrical stems prescribe a tensile stress pattern in the epidermis, with the maximal tensile stress in the transverse direction (following the same Laplace-Young law as in the ellipsoidal protoplast discussed above). This can be revealed with cell-cell adhesion mutants: longitudinal cracks are consistent with transverse tension (Verger et al., 2018). Similar cracks can also emerge when the balance between epidermal resistance is reduced and growth of inner tissues is enhanced (Asaoka et al., 2021).

### *In vitro* animal models for exploring the role of geometry

Tissue shaping is a multi-scale process, from subcellular mechanical interactions through to feedback from system boundary constraints. Disentangling mechanical and geometric factors underlying morphogenesis can be challenging, especially *in vivo*. Recent advances in organoids – *in vitro* approaches to replicate elements of organs in the lab environment – are providing opportunities to disentangle how mechanics and geometry impact tissue shaping.

Organoids are derived from stem cells and self-assemble into 3D structures that (at least partially) mimic *in vivo* cell types and organ structure. Organoids have been created for mimicking organs ranging from the gut (Barker et al., 2007; Sato et al., 2009) to the brain (Villalba et al., 2021). A key advantage of organoids is that tuning its boundary shape is (comparatively) simpler than *in vivo* perturbation of organ shapes. Therefore, the role of geometry in organ formation can be investigated in a more systematic manner.

Formation of gut organoids has been shown to be sensitive to the initial system size (Gjorevski et al., 2022). Feedback between geometry of the surrounding matrix and patterning guides crypt formation. This feedback is, in part, mediated by the mechanosensitive YAP pathway (Dupont et al., 2011). As cell crowding increases in geometrically confined organoids, YAP activity is decreased in a geometry-dependent manner (Maimets et al., 2026). Similar behaviour has recently been observed in an organoid model of neuromesodermal progenitors (NMPs), also known as neuruloids (Rito et al., 2025).

Organoid systems can be used to explore the interplay between geometry and system patterning (Kicheva and Briscoe, 2023; Warmflash et al., 2014). In the neuruloid system, the geometry determines the spatial pattern of symmetry breaking into distinct mesoderm-like and neural-like domains (Loo et al., 2026). A simple reaction-diffusion model of patterning was able to accurately predict the emerging pattern of fate domains under different conditions by accounting for altered boundary constraints (Fig. 5C,D).

The combination of micropatterning of domains with organoids offers accessibility to probe how the mechanical state of tissues changes in different geometries (Valet et al., 2022). For example, micropatterning domains of laminin can define distinct domains for organoids (Rito et al., 2025). Organoids of optic cup formation have proven to be a powerful system for such investigation (Okuda et al., 2018). Mechanical feedback, which depends on the local tissue curvature, drives folding and formation of the optic cup morphology and is able to replicate the observed regions of positive and negative curvature during optic cup formation. In particular, bending of the neural retina depends on the emergence of spontaneous curvature in the system; the system geometry appears to be instructive for cup shaping.

Interestingly, mechanical feedback from embedded geometric constraints does not only shape the tissue, but also plays an important role in ensuring robust morphogenesis. In an intestinal organoid system, feedback between fluid pressure and cell-scale tension determined how cells adapted to the changing local environment during morphogenesis. This feedback enabled the system to correct for errors in growth, ensuring that the final tissue form was reproducible (Xue et al., 2025).

Of course, organoids have limitations; they are simplified models of *in vivo* organ-derived tissues that only replicate certain elements of organogenesis, e.g., they typically lack vasculature. Further, cell fate patterning in organoids does not necessarily correspond to the *in vivo* process it seeks to recapitulate. Nonetheless, these new *in vitro* systems are revealing insights into how geometry impacts morphogenesis. In particular, it is becoming increasingly clear that geometry is not merely a passive guide to morphogenesis, but is also essential for its robustness; i.e. that local geometry provides feedback during morphogenesis that can ensure that the process occurs reproducibility. As an exciting potential future avenue, cardioids provide a model system to explore size and boundary dependency in heart formation (Hofbauer et al., 2021; Schmidt et al., 2023).

## Theoretical approaches to understand how geometry informs morphogenesis

In this final section, we outline recent developments in models of biological tissues that are revealing how geometry and morphogenesis intersect to reliably build specific tissue morphologies. We focus on two areas which have been driven by theoretical advances: geometric constraints in mechanochemical feedback and the impact of system curvature on cell and tissue shape.

### Boundary feedback into growth and size control

Returning to the process of *Drosophila* germband elongation, tissue elongation occurs near the surface of the closed, ellipsoidal-like embryo. As the germband elongates, the neighbouring dorsal amnioserosa stretches in response. By modelling both the actively elongating lateral ectoderm and passively stretching amnioserosa within the confining embryonic geometry, patterns of tissue flow could be reliably simulated (as discussed previously; Brauns et al., 2024; Claussen et al., 2024).

Recent work has integrated topological control of cell alignment within different geometric constraints (Guillamat et al., 2026). A thin-shell model of a tissue in different geometries incorporating the pre-defined topological constraints was able to accurately predict the 3D shape of the resulting tissue. The tuning of geometry with topology within a predictive model opens exciting new avenues for programming specific tissue shapes (Box 2).

Box 2. Modelling the impact of geometry on morphogenesis This review principally focuses on the instructive role that geometry plays during development. We briefly outline here the theoretical approaches that have been used to explore this challenge.

**L-systems.** Lindenmayer (L)-systems describe theoretical systems that offer sets of repeatable rules that define local system growth. Cell states are defined through a ‘grammar’ that defines their state and neighbourhood, avoiding the use of indexing. The topological arrangement of cells can be easily updated upon cellular processes such as division. This grammar structure makes them amenable to computational modelling, and they have proven particularly successful in describing plant morphogenesis (Lindenmayer, 1968a,b; Prusinkiewicz, 2025). Importantly, the output of L-systems can be mapped onto geometric representations, enabling predictions for tissue formation to be linked to experimental measurements.

**Thin-shell models.** These describe the behaviour of thin sheets of confluent cells (typically epithelia). It assumes the sheets are continuous, thin (i.e. much larger in the plane than in depth) and can be approximated by elastic or viscoelastic dynamics. While the sheets are assumed to be 2D, they can bend, fold and buckle, making this a powerful approach for understanding the importance of geometry in morphogenetic processes (Lou et al., 2023).

**Vertex models.** Suitable for understanding highly confluent tissues (such as epithelia), each cell is represented as a polygon. The sides of the polygons correspond to the cell membrane. The movement of the vertices (corners) in the vertex model are then governed by biophysical principles, such as surface area constraints (Fletcher et al., 2014). These rules can be easily adapted, enabling vertex models to be applied to a broad range of 2D systems. Vertex models are relatively straightforward to simulate (at least in 2D), and can incorporate geometrically diverse situations, including highly curved systems.

**Active foam models.** While vertex models can describe highly confluent tissues, cell interactions within many biological systems are more complex. For example, during formation of somites, the presence of extracellular space is crucial in determining the system behaviour (Mongera et al., 2018). The formulation of active foam models is similar to vertex models, but they can incorporate more details, such as extracellular spaces and fluctuations of cell edges (Kim et al., 2021).

### The role of curvature in determining tissue shape

Epidermal curvature can be an instructive cue in pressurised systems like plants. Indeed, as discussed above, the Laplace-Young law relates curvature and tension. Organ surfaces can thus act as ‘mechanical waveguides’ (Lintilhac, 2026), directing internal stress fields, the orientation of which correlates with cell behaviour; for example, the placement of new cell walls during division (Louveaux et al., 2016). This links surface curvature to tissue patterning. Recently, the stress patterns in leaf epidermis have been shown to impact the division plane of guard cells (stomata), leading to large-scale co-alignments (Serra et al., 2026). In that case, the final division plane is a compromise between cell geometry cues (the shortest plane) and the tissue geometry-driven stress pattern. There have been extensive reviews on the molecular players linking curvature (e.g. the BAR proteins; Sitarska et al., 2023) and mechanical response of cells (e.g. through the mechanotransductive YAP pathway; Panciera et al., 2017). Here, we focus on the cell- to tissue-scale processes shaping tissues.

The shoot apical meristem (SAM) illustrates the patterning role of tissue curvature (Burian et al., 2013). The SAM is a dome-shaped structure, from the side of which lateral aerial organs (leaves, flowers) emerge. At the tip of the dome, the geometry is locally isotropic, also matching dynamic and rather disorganised cortical microtubules and unpredictable cell division plane orientations. This also corresponds to the central zone, where the stem cell niche is located. At the base of the dome, the geometry is closer to a cylinder. Cortical microtubules and cell divisions planes are increasingly transverse, matching the shape-derived maximal tensile stress (in accordance with the Laplace-Young law). This also corresponds to the peripheral zone, where growth is increased and lateral organs can emerge. Interestingly, auxin patterning, which determines the position of lateral organs, has also been related to geometry (Heisler et al., 2010; Vernoux et al., 2021). The boundary between the emerging organ and the meristem is a saddle-shaped domain, with negative Gaussian curvature (Box 1). This prescribes highly anisotropic tensile stress direction, with very stable cortical microtubule orientation (Hamant et al., 2008). In this domain, cells divide along the longest plane too, matching tissue-shape-derived stress rather than cell-shape-derived stress (Louveaux et al., 2016). Furthermore, as the organ grows, deep boundary cells are progressively squeezed between the organ and meristem. Those cells experience negative growth, i.e. shrinkage, as water is drained out by the emerging organ, in synergy with local compression. Consistently, a molecular signature of water stress can be recorded in that domain. In other words, water flux becomes a morphogen *lato sensu* (in the Turing sense of ‘morphogen’ signals that induce patterns through reaction-diffusion systems), and this flux is indirectly the result of tissue curvature (Alonso-Serra et al., 2024).

**Fig. 6.**
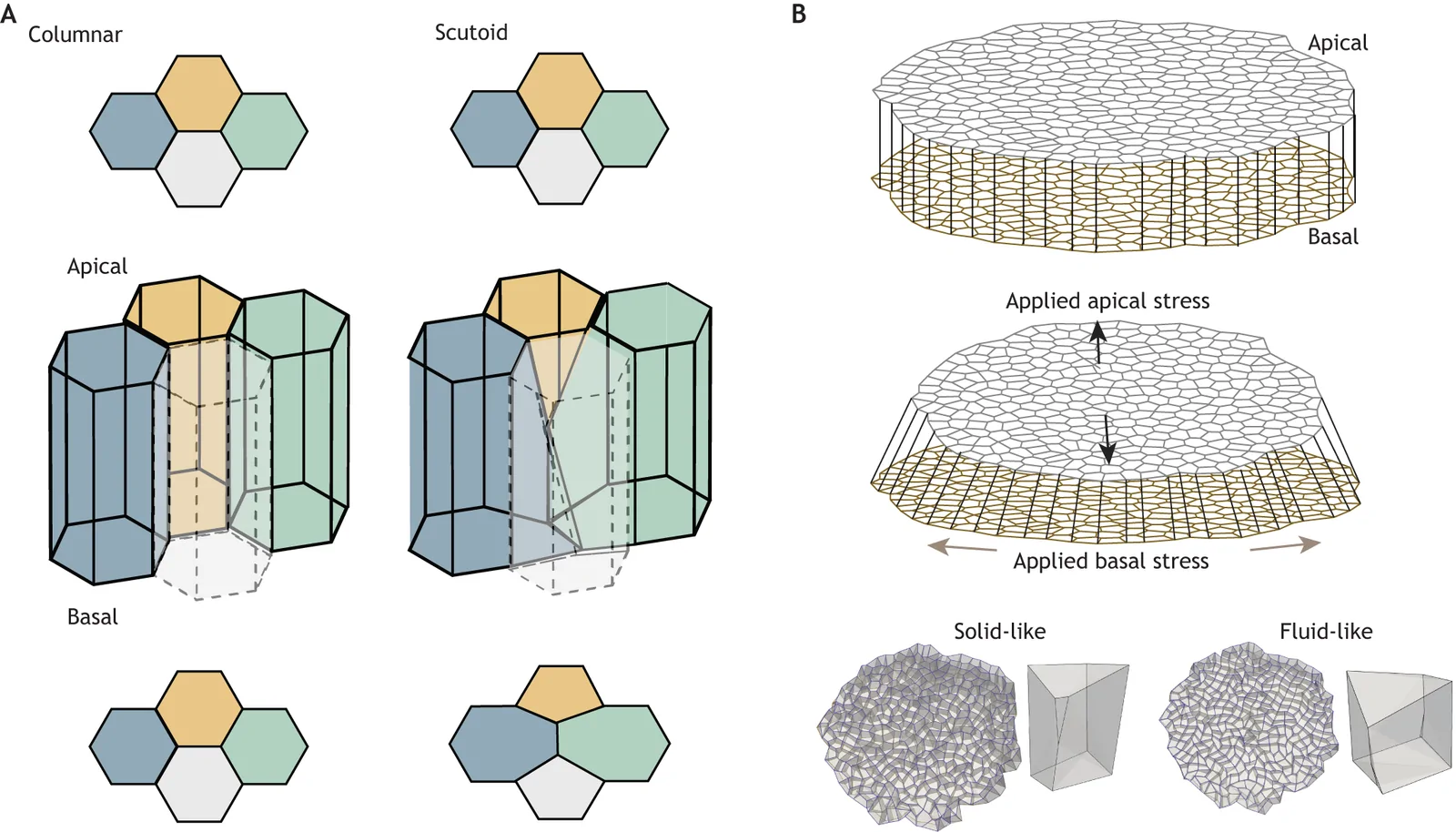
**Boundaries alter cell packing.** (A) Example of columnar and scutoid cell packing, the latter occurring more frequently in curved geometries. (B) Cell shape dependence with anisotropic stress depends on the cell properties, such as tension membrane and volume. Top: an unconstrained 3D tissue. Middle: the change in shape with anisotropic stress (as can occur for geometrically confined tissues). Bottom: the tissue and cell shape with applied stress in solid-like and fluid-like parameter regimes.

In animal epithelial cells, geometric constraints can initiate cell shape changes from apical-to-basal surfaces (Fig. 6A), which leads to a cell shape known as ‘scutoid’ (Gomez-Galvez et al., 2018). Scutoids have different neighbours at apical and basal surfaces, enabling them to remain confluent cell packing despite the geometric constraints. Such behaviour is seen across a range of systems, from epithelial organisation in the early sea star embryo to *Drosophila* (including tube formation in the highly curved salivary gland and cell packing in the embryo anterior) (Barone et al., 2024; Gomez-Galvez et al., 2018; Rupprecht et al., 2017; Sun et al., 2017).

Biophysical models of cell monolayers in curved environments have been developed for both highly curved geometries and cells with large apical-basal extent (Gomez-Galvez et al., 2018; Lou et al., 2023; Rupprecht et al., 2017). An advantage of these theoretical approaches is that they can be generalised to provide predictions for a range of systems and system properties, such as different cell mechanical properties (Fig. 6B) (Lou et al., 2026). One such example is the emergence of scutoids in curved confluent tissues (Fig. 6A). Scutoids are a consequence of strain anisotropy across the cell, which can be generated by large curvature gradients, resulting in cell tilting and subsequently scutoid geometries, as is the case for cell packing in the early *Drosophila* embryo (Lou et al., 2026; Rupprecht et al., 2017). In this case, the epithelial cells begin as columnar but undergo shape changes as they extend along the apical-basal axis and thereby experience larger constraint from the local curvature of the surface. Strain anisotropy can also be generated via other mechanisms, such as differences in pressure across the tissue in regions with high curvature, as appears to be the case for cell packing in narrow epithelial tubes (Gomez-Galvez et al., 2018). How cells arrange in three dimensions in response to geometric constraints is the result of competition between cell pressure, the local curvature and cell adhesion.

Given that morphogenesis comprises distinct processes – e.g. cell division, branching, growth – one approach for modelling organ growth is through theoretical approaches that accommodate the integration of multiple parameters, such as Lindenmayer (L)-systems (Box 2). L-systems traditionally operate only in flat (Euclidean) 2D or 3D space. Many real organisms, however, develop on curved, non-flat surfaces – such as leaf veins, pollen tubes, tooth patterns or signals diffusing across curved tissues. To address this, one can extend L-systems into non-Euclidean geometry by incorporating concepts from differential geometry (Abelson and DiSessa, 1986). This allows the simulation of mathematical and biological forms growing directly on curved surfaces. This approach has recently been further generalised to model branching systems growing within non-uniform or intrinsically curved substrates (Godin and Boudon, 2025).

Finally, boundary constraints can induce a phenomenon called geometric frustration, which refers to stresses built up when the preferred geometry of the tissue is incompatible with the surrounding boundary constraints (Abergel, 2025). More specifically, geometric frustration means that there are certain positions at which the tissue cannot adapt to changes in the local curvature. As a result, large stresses build up at these points, leading to abrupt shape changes. Recent work has shown that rose petal shaping is a consequence of such frustration (Zhang et al., 2025). Exploding fruits, e.g. in Cardamine, also reflect such geometric frustration, through rapid rupture, providing long-range seed dispersal (Cullen and Hay, 2024).

From the discussions above, we see that geometric constraints can be crucial for generating shapes through a variety of mechanisms. In this section, we have largely focused on models that are assumed to be in or close to steady state and in relatively simple spatial domains; i.e. the system dynamics are relatively unimportant in the tissue shaping and smoothly curving surfaces. Of course, this is often an oversimplification, and a key next stage is extending modelling of tissue shape formation to more dynamic systems (e.g. using active foam approaches; Kim et al., 2021) or with more complex geometry (e.g. the intestinal crypt).

## Conclusions

Recent years have seen a marked increase in our understanding of how geometry impacts morphogenesis across both the animal and plant kingdoms. In this Review, we have highlighted situations where geometry is instructive for tissue shaping, and that surrounding tissues or matrices are not simply a passive boundary or passive source of signalling molecules. The impact of geometry is seen across different levels. For example, in tube formation (as is common in plants) the curvature of the tube itself provides feedback to the developing organ. Developing organs can alter shape as they develop, such as follicle elongation of the *Drosophila* oocyte (Fig. 2A), and these geometric changes feedback into the developmental programme. It is important to also consider the geometry of constraining tissues and boundaries to the developing organ. For example, during *Drosophila* early development, differences in curvature around the embryo posterior impact shaping of emerging organs (Fig. 2C). Finally, developing organs can change the shape of the surrounding environment, such as in root growth through soil or myotome formation in zebrafish (Fig. 4A-C). These examples emphasise the role of geometry in ensuring robust organ development.

Feedback with the boundary is driven through mechanical interactions and can depend on finely tuned viscosity through changes in the ECM. Of course, as tissues change shape, they can dynamically alter the extent of signalling inputs from the boundaries and such factors are now being considered in models of morphogenesis. Advances in *in vitro* models, such as organoid systems and patterned surfaces (Fig. 5A-D), and better *in vivo* perturbation approaches, are enabling us to dissect how geometry instructs morphogenesis. Yet, as we have highlighted throughout this Review, many open questions remain.

The geometry defined by boundaries plays an important role in shaping tissues, which can be mediated by finely tuned adhesion interactions. Of course, we have only scratched the surface here and there are a host of other local-scale processes, such as apical-basal polarity (Almasoud et al., 2026), dynamic matrix metalloprotease expression (Ku et al., 2023; Page-McCaw et al., 2007), hydraulic pressure (Munjal et al., 2021) and signalling–receptor interactions (Brown, 2011) that are important in determining tissue shape and responding to local geometric constraints. Further, apoptosis (Levayer et al., 2016), cell crowding (Devany et al., 2023) and hydrostatic pressure (Chugh et al., 2022) are processes dependent on the physical environment that can be active drivers of tissue shaping. In recognition of this, we have focused on the role of geometry itself instead of specific molecular mechanisms. As our understanding of boundary effects develops, it will be exciting to generate more integrated models that represent the multi-scale, multi-component factors that shape developing organs.

This Review has focused on geometry at cellular and tissue scales. Yet, subcellular mechanisms can recognise curved membrane geometry, as is known for the Bar-domain proteins (Simunovic et al., 2015). A new hotspot in plant research relates to geometry at subcellular scales: cell edges. In the past decade or so, several proteins have been shown to exhibit preferential polarities along certain cell edges (i.e. the lines where two cell faces meet). For example, the microtubule regulator CLASP localises to certain edges and is thought to prevent microtubule catastrophe, notably when the edge angle is sharp (a property relating to microtubule persistence length; Ambrose et al., 2011). Endomembrane GTPase RAB-A5c also localises to certain cell edges and is thought to contribute to the early prescription of growth anisotropy in lateral roots, before cortical microtubules alignment (Kirchhelle et al., 2016, 2019). Soseki proteins localise to certain corners of the cells, with protein-specific polarities (Yoshida et al., 2019). There is increasing evidence that such polarities depend on mechanical properties (Piepers et al., 2025 preprint). These results take the question of geometric boundaries to the subcellular micrometric scale, while not reaching the molecular nanometric scale. It will be interesting to see how biological systems integrate instructive geometric cues across multiple scales.

As briefly noted above, tuning of geometry provides a tantalising prospect for understanding the evolution of complex form (Boutillon et al., 2024; Huang et al., 2026). Rather than rewiring genetic networks or evolving new mechanical processes, altering the system geometry can have a major impact on tissue shape and size (Swalla, 2006). These ideas have recently been touched upon in relation to ridge formation in the skin (Thompson et al., 2026) and emergence of multicellularity in archaea (Rados et al., 2025). The use of micropatterned organoids provides a potential route to explore these concepts more deeply (Anlas and Trivedi, 2021; Steventon et al., 2021; Xu et al., 2025 preprint).

Overall, in this Review we have provided a broad overview of how geometry can be instructive for development. Though specific mechanisms vary considerably, we have highlighted that geometry and morphogenesis are tightly interlinked in both animal and plant model systems. With increasingly powerful experimental and theoretical techniques we are at an exciting stage where we can truly dissect the role of geometry in morphogenesis.
